# Supplementary material for: Sequential intrahost evolution and onward transmission of SARS-CoV-2 variants
Source: Nat Commun. 2023 Jun 3;14:3235. doi: 10.1038/s41467-023-38867-x (PMC10239218; doi:10.1038/s41467-023-38867-x)
Supplement: Supplementary file 1 — Supplementary Information [file 41467_2023_38867_MOESM1_ESM.pdf]

SUPPLEMENTARY INFORMATION FOR

# Sequential intrahost evolution and onward transmission of SARS-CoV-2 variants

Ana S. Gonzalez-Reiche[1], Hala Alshammary[2,3], Sarah Schaefer[4], Gopi Patel[4], Jose Polanco[2,3], Juan Manuel Carreño [2,3], Angela A. Amoako[2,3], Aria Rooker[2,3], Christian Cognigni[2,3], Daniel Floda[1], Adriana van de Guchte[1], Zain Khalil[1], Keith Farrugia[1], Nima Assad[5], Jian Zhang[1], Bremy Alburquerque[1,5], PARIS/PSP study group[2,3], Levy Sominsky[2,3], Charles Gleason[2,3] Komal Srivastava[2,3], Robert Sebra[1,6,7] , Juan David Ramirez[8,9], Radhika Banu[8], Paras Shrestha[8], Florian Krammer[2,3,8], Alberto Paniz-Mondolfi[8], Emilia Mia Sordillo[8 †], Viviana Simon[2,3,4,8,10, †], Harm van Bakel[1,2,6,†]

1. Department of Genetics and Genomic Sciences, Icahn School of Medicine at Mount Sinai, New York, NY 10029, USA
2. Department of Microbiology, Icahn School of Medicine at Mount Sinai, New York, NY 10029, USA
3. Division of Infectious Diseases, Department of Medicine, Icahn School of Medicine at Mount Sinai, New York, NY 10029, USA
4. Center for Vaccine Research and Pandemic Preparedness (C-VaRPP), Icahn School of Medicine at Mount Sinai, New York, NY 10029, USA
5. Graduate School of Biomedical Sciences, Icahn School of Medicine at Mount Sinai, New York, NY 10029, USA
6. Icahn Genomics Institute, Icahn School of Medicine at Mount Sinai, New York, NY 10029, USA
7. Black Family Stem Cell Institute, Icahn School of Medicine at Mount Sinai, New York, NY, 10029, USA The Global Health and Emerging Pathogens Institute, Icahn School of Medicine at Mount Sinai, New York, NY 10029, USA
8. Department of Pathology, Molecular, and Cell-Based Medicine, Icahn School of Medicine at Mount Sinai, New York, NY 10029, USA
9. Centro de Investigaciones en Microbiología y Biotecnología-UR (CIMBIUR), Facultad de Ciencias Naturales, Universidad del Rosario, Bogotá, Colombia.
10. The Global Health Emerging Pathogens Institute, Icahn School of Medicine at Mount Sinai, New York, NY 10029, USA.

Corresponding authors/senior authors:

† Dr. Emilia Mia Sordillo (Emilia.Sordillo@mountsinai.org), Dr. Viviana Simon (viviana.simon@mssm.edu), and Dr. Harm van Bakel (harm.vanbakel@mssm.edu)

**This PDF contains:**

Supplementary Figures 1 to 4.
Supplementary Tables 1 to 3.

**Supplementary Figure 1: Overview of genome-wide SARS-CoV-2 mutations found in diagnostic nasal biospecimen.** The mutations present across the complete SARS-CoV-2 genome are shown. Nucleotide substitutions observed in sequential specimens obtained from patient 1 (P1) with prolonged infection with BA.1 and forward transmission cases (P2, P3 and P4). There is accumulation and fixation of new SNVs in the spike region in P1. The same constellation of mutations was subsequently detected in three documented transmission cases (P2, P3 and P4) and in two sequences from GISAID (EPI_ISL_11628400 and EPI_ISL_11696379). The only shared synonymous SNV outside of the spike (ORF1a:T6001C) is indicated with an arrow. The number of days since the first positive test in P1 is shown on the left, with the number of days after the first positive test for each patient between brackets. The BA.1 substitutions are shown in blue and novel substitutions are shown in red, relative to the reference genome sequence NC_045512.2. "N" represents ambiguous bases with coverage below the base-calling threshold of 10X. The minimum required sequence read coverage for minor variant calling is 100X.

## Supplementary Figure 1

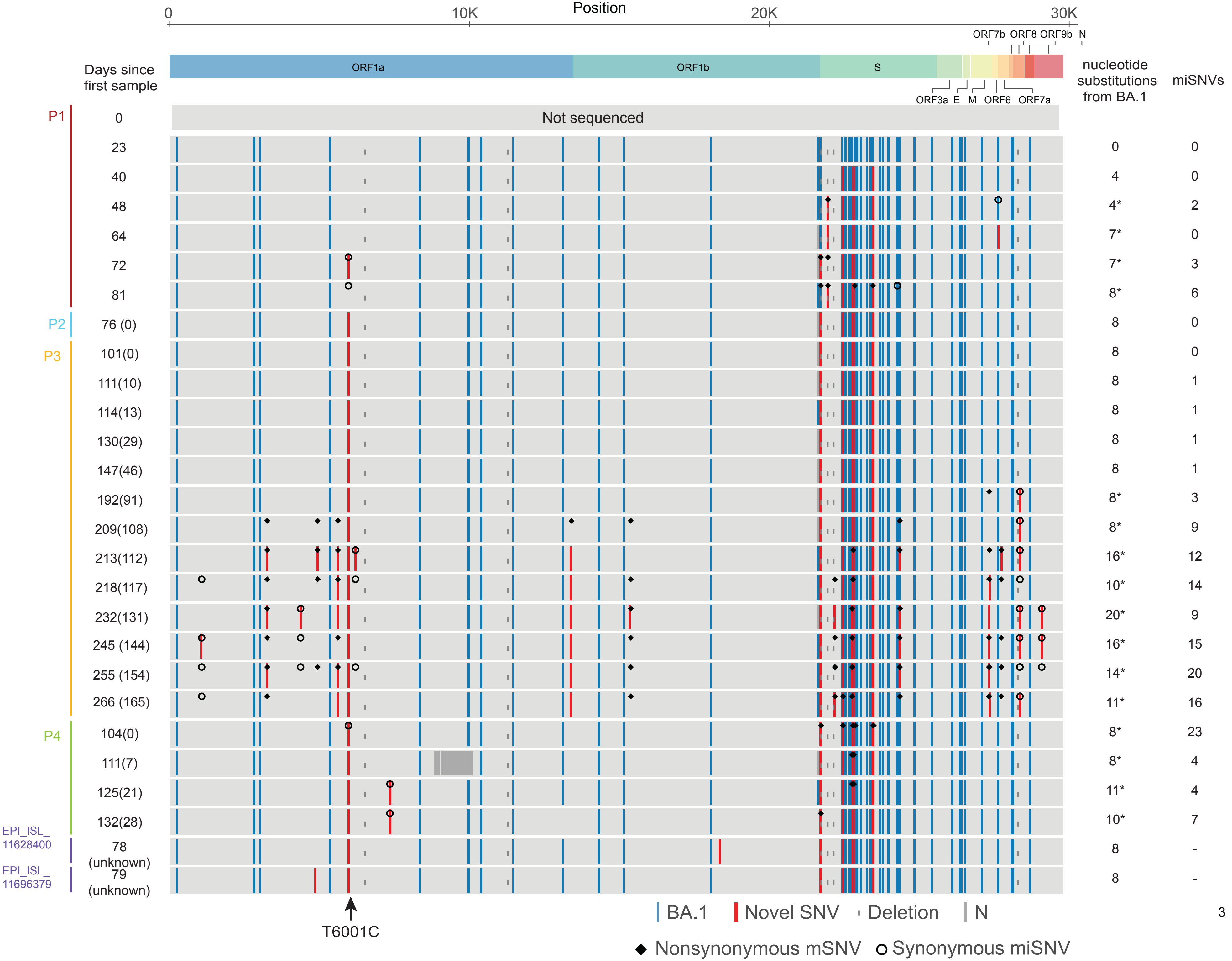

**Supplementary Figure 2: Summary of SARS-CoV-2 mutations within the spike gene in minority viral populations.**

The distribution of minority intrahost single nucleotide variants (miSNVs) for positions that were fixed over the course of infection in the index and transmission cases are shown indicated with asterisks. Single nucleotide variants are colored by strain. The dotted bars indicate positions with coverage below the threshold of 100X coverage for calling miSNVs. The positions with minority variants present at frequencies > 0.1 are indicated with diamonds for their respective samples.

## Supplementary Figure 2

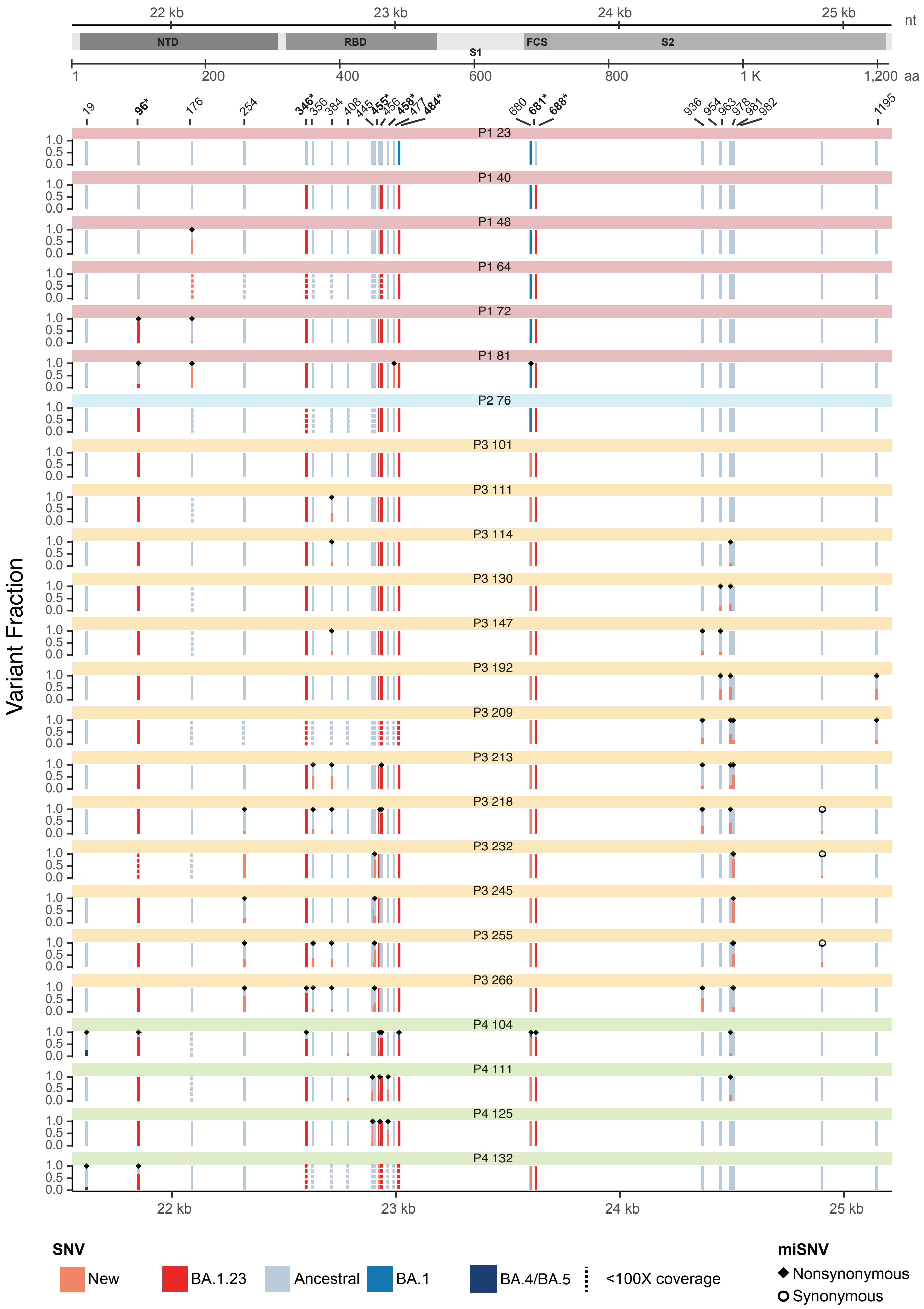

**Supplementary Figure 3: Summary of genome-wide SARS-CoV-2 mutations in minority viral populations for patient P3**

Similar to Supplementary Fig. 2 but showing the distribution of nucleotide variants below the consensus level observed in one or more specimens from P3. Single nucleotide variants (SNVs) are colored by strain. The dotted bars indicate positions with coverage below the threshold of 100X coverage for calling miSNVs. The positions with minority variants at frequencies > 0.1% are indicated with triangles (nonsynonymous) or circles (synonymous) for their respective samples.

## Supplementary Figure 3

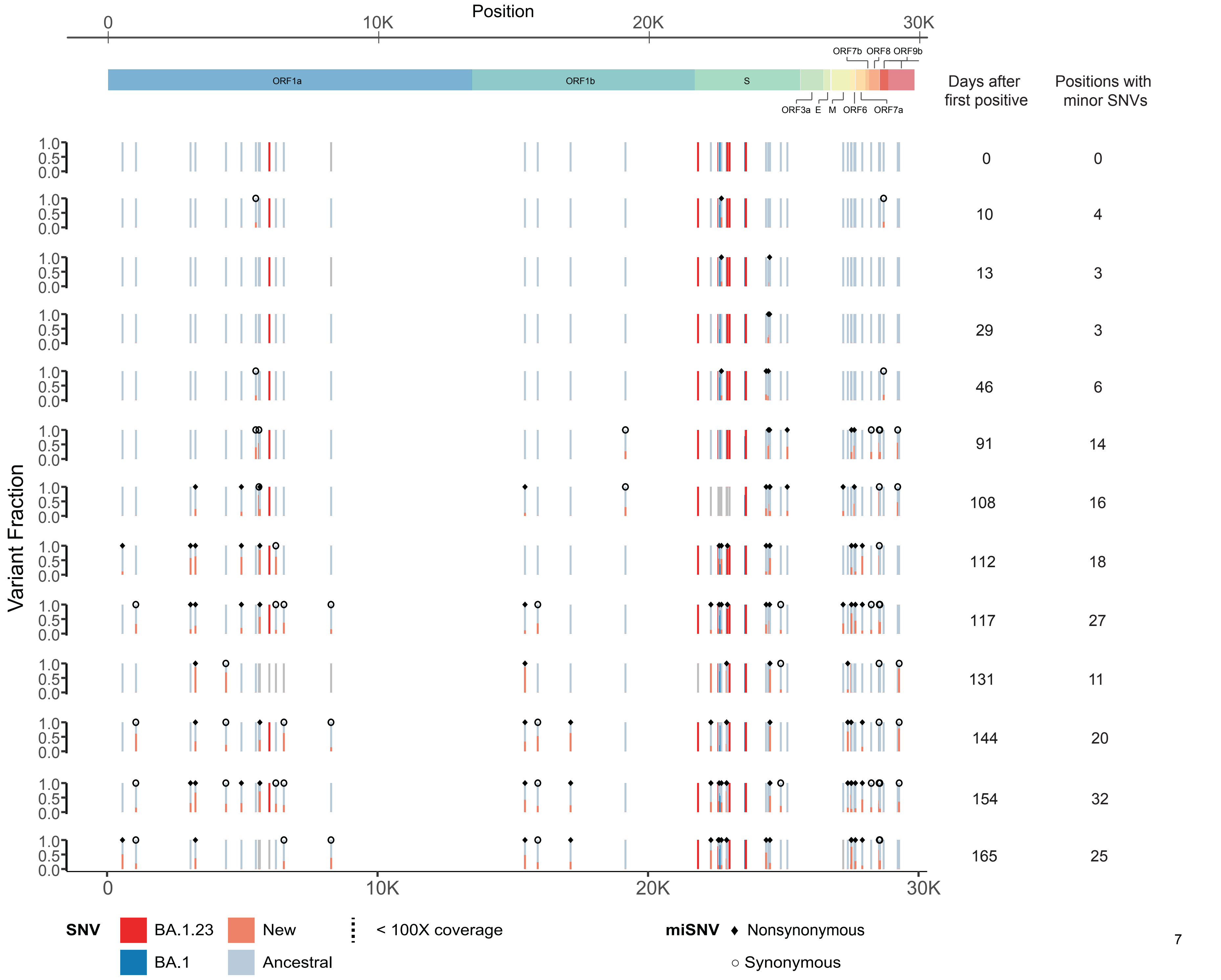

**Supplementary Figure 4: Summary of SARS-CoV-2 mutations within the Spike gene in minority viral populations for patient P3**

Similar to Supplementary Fig. 3 but showing minority mutations in the Spike gene region. Single nucleotide variants (SNVs) are colored by strain. The dotted bars indicate positions with coverage below the threshold of 100X coverage for calling minor SNVs (miSNVs). The positions with minority variants at frequencies > 0.1 are indicated with triangles for their respective samples.

## Supplementary Figure 4

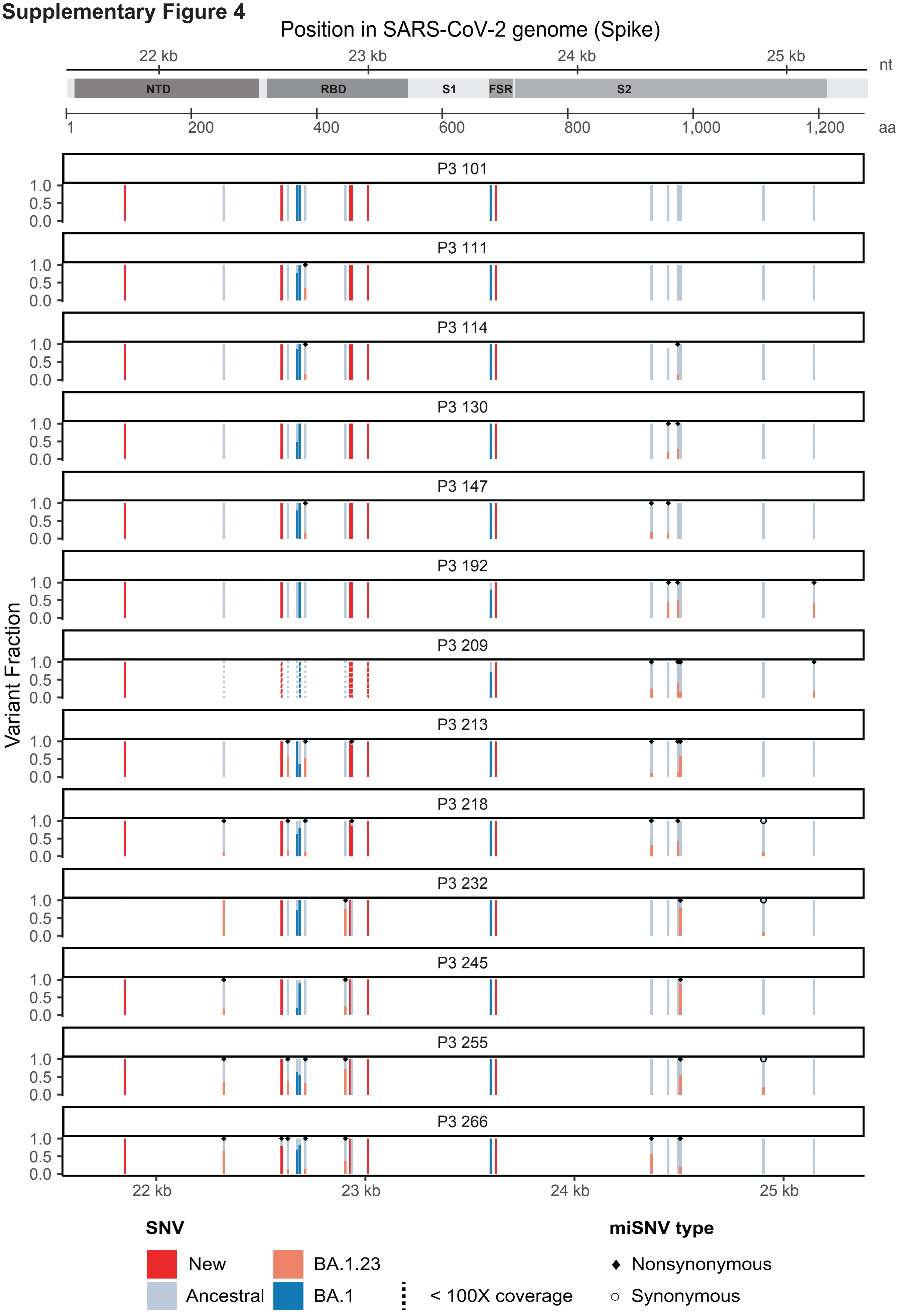

**Supplementary Table 1**. Overview of the two serum panels used in this study

| | | | | |
|---|---|---|---|---|
| Panel 1 (Three mRNA vaccine doses) | Total # of participants selected | 9 | | |
| | Age average (min-max) | 59.7 (50-69 years) | | |
| | Sex | Female | | 8 |
| | | Male | | 1 |
| | Total # Sera | 18 | | |
| | Days between sample collection and vaccine dose | Before | Average | 19 |
| | | | Range | 0-38 |
| | | After | Average | 31 |
| | | | Range | 14-59 |
| Panel 2 (Omicron BA.1 Breakthroughs) | Total # of participants selected | 11 | | |
| | Age average (min-max) | 43.6 (18-69 years) | | |
| | Sex | Female | | 7 |
| | | Male | | 4 |
| | Total # Sera | 22 | | |
| | Days between sample collection and infection | Before | Average | 11 |
| | | | Range | 0-27 |
| | | After | Average | 27 |
| | | | Range | 15-37 |

**Supplementary Table 2**. Detailed characteristics of the two serum panels used in this study

| Participant ID | Age Bracket | Sex | Time points included in this study |
|---|---|---|---|
| BO-1 | 60-69 | Male | Pre-Booster<br>Post Booster |
| BO-2 | 50-59 | Female | Pre-Booster<br>Post Booster |
| BO-3 | 50-59 | Female | Pre-Booster<br>Post Booster |
| BO-4 | 60-69 | Female | Pre-Booster<br>Post Booster |
| BO-5 | 60-69 | Female | Pre-Booster<br>Post Booster |
| BO-6 | 50-59 | Female | Pre-Booster<br>Post Booster |
| BO-7 | 50-59 | Female | Pre-Booster<br>Post Booster |
| BO-8 | 60-69 | Female | Pre-Booster<br>Post Booster |
| BO-9 | 60-69 | Female | Pre-Booster<br>Post Booster |
| BABI-1 | 30-39 | Female | Pre-infection, Boosted<br>Post-infection, Boosted |
| BABI-2 | 40-49 | Male | Pre-infection, Boosted<br>Post-infection, Boosted |
| BABI-3 | 40-49 | Male | Pre-infection, Unboosted<br>Post-infection, Unboosted |
| BABI-4 | 40-49 | Female | Pre-infection, Unboosted<br>Post-infection, Unboosted |
| BABI-5 | 30-39 | Female | Pre-infection, Unboosted<br>Post-infection, Unboosted |
| BABI-6 | 30-39 | Female | Pre-infection, Boosted<br>Post-infection, Boosted |
| BABI-7 | 60-69 | Female | Pre-infection, Boosted<br>Post-infection, Boosted |
| BABI-8 | 18-29 | Male | Pre-infection, Boosted<br>Post-infection, Boosted |
| BABI-9 | 60-69 | Male | Pre-infection, Boosted<br>Post-infection, Boosted |
| BABI-10 | 40-49 | Female | Pre-infection, Boosted<br>Post-infection, Boosted |
| BABI-11 | 40-49 | Female | Pre-infection, Boosted<br>Post-infection, Boosted |
| Patient 2 described in this paper | 60-69 | Female | Pre-infection, Unboosted<br>Pre-infection, Boosted<br>Post-infection, Boosted<br>Post-infection, Boosted |
