# Supplementary material for: Sequential intrahost evolution and onward transmission of SARS-CoV-2 variants
Source: Nat Commun. 2023 Jun 3;14:3235. doi: 10.1038/s41467-023-38867-x (PMC10239218; doi:10.1038/s41467-023-38867-x)
Supplement: Supplementary file 3 — Reporting Summary [file 41467_2023_38867_MOESM3_ESM.pdf]

nature portfolio

Corresponding author(s): Harm van Bakel, Viviana Simon, Emilia Mia Sordillo

Last updated by author(s): Apr 12, 2023

Reporting Summary

Nature Portfolio wishes to improve the reproducibility of the work that we publish. This form provides structure for consistency and transparency in reporting. For further information on Nature Portfolio policies, see our Editorial Policies and the Editorial Policy Checklist.

Statistics

For all statistical analyses, confirm that the following items are present in the figure legend, table legend, main text, or Methods section.

n/a	Confirmed	
☐	☒	The exact sample size (*n*) for each experimental group/condition, given as a discrete number and unit of measurement
☐	☒	A statement on whether measurements were taken from distinct samples or whether the same sample was measured repeatedly
☐	☒	The statistical test(s) used AND whether they are one- or two-sided<br>*Only common tests should be described solely by name; describe more complex techniques in the Methods section.*
☒	☐	A description of all covariates tested
☐	☒	A description of any assumptions or corrections, such as tests of normality and adjustment for multiple comparisons
☐	☒	A full description of the statistical parameters including central tendency (e.g. means) or other basic estimates (e.g. regression coefficient) AND variation (e.g. standard deviation) or associated estimates of uncertainty (e.g. confidence intervals)
☐	☒	For null hypothesis testing, the test statistic (e.g. *F*, *t*, *r*) with confidence intervals, effect sizes, degrees of freedom and *P* value noted<br>*Give P values as exact values whenever suitable.*
☒	☐	For Bayesian analysis, information on the choice of priors and Markov chain Monte Carlo settings
☒	☐	For hierarchical and complex designs, identification of the appropriate level for tests and full reporting of outcomes
☒	☐	Estimates of effect sizes (e.g. Cohen's *d*, Pearson's *r*), indicating how they were calculated

Our web collection on statistics for biologists contains articles on many of the points above.

Software and code

Policy information about availability of computer code

Data collection	Next-generation sequencing data was collected with the standard tools provided by Illumina.
Data analysis	Open source code used for data analysis includes: vRAPID, a custom SARS-CoV-2 genome assembly pipeline (https://github.com/BakelLab/vRAPID, https://github.com/mjsull/COVID_pipe); MASH v2.3; SARS-CoV-2 phylogenetic reconstruction and genotyping software: Nextstrain v11 (https://github.com/nextstrain/ncov), Nextclade CLI v3.2 (2021-11-04); PANGO-v1.8 (pangolin v3.1.17, pangoLEARN v.2022-04-22), IQ-TREE v.2.1.2. RIPPLES (v.0.0.1), UShER (v0.6.1); Statistical analysis of serological assays: Prism 9 (GraphPad).

For manuscripts utilizing custom algorithms or software that are central to the research but not yet described in published literature, software must be made available to editors and reviewers. We strongly encourage code deposition in a community repository (e.g. GitHub). See the Nature Portfolio guidelines for submitting code & software for further information.

Data

Policy information about availability of data

All manuscripts must include a data availability statement. This statement should provide the following information, where applicable:

- Accession codes, unique identifiers, or web links for publicly available datasets
- A description of any restrictions on data availability
- For clinical datasets or third party data, please ensure that the statement adheres to our policy

Complete genome sequences for the viral isolates cultured from nasal swabs (BA.1 and BA.1.23) are available in GenBank (accession numbers ON220548 [https://

www.ncbi.nlm.nih.gov/nuccore/ON220548], ON220539 [https://www.ncbi.nlm.nih.gov/nuccore/ON220539], ON196014 [https://www.ncbi.nlm.nih.gov/nuccore/ON196014], ON193425 [https://www.ncbi.nlm.nih.gov/nuccore/ON193425], ON220529 [https://www.ncbi.nlm.nih.gov/nuccore/ON220529], ON220571 [https://www.ncbi.nlm.nih.gov/nuccore/ON220571], ON220533 [https://www.ncbi.nlm.nih.gov/nuccore/ON220533], ON934538 [https://www.ncbi.nlm.nih.gov/nuccore/ON934538], ON934577 [https://www.ncbi.nlm.nih.gov/nuccore/ON934577], ON934030 [https://www.ncbi.nlm.nih.gov/nuccore/ON934030], ON934607 [https://www.ncbi.nlm.nih.gov/nuccore/ON934607], ON854465 [https://www.ncbi.nlm.nih.gov/nuccore/ON854465], ON619382 [https://www.ncbi.nlm.nih.gov/nuccore/ON619382]). RNA-seq data are available in the Sequence Read Archive (SRA), submission SUB12865927 under BioProject number PRJNA623586 [https://www.ncbi.nlm.nih.gov/sra/?term=PRJNA623586] (BioSample accession numbers SAMN33273632- SAMN33273655).

Research involving human participants, their data, or biological material

Policy information about studies with human participants or human data. See also policy information about sex, gender (identity/presentation), and sexual orientation and race, ethnicity and racism.

Reporting on sex and gender	Reference sera for neutralization assays collected before and after SARS-CoV-2 booster vaccination (N=9 participants) as well as before and after Omicron BA.1 break-through infections (N=11 participants) were selected for testing in this study. In addition, we had access to banked sera from one of the forward transmission cases (P2). All 20 PARIS participants provided written consent prior to study participation and data collection (IRB-20-03374).<br><br>Samples from before and after the infection with BA.1.23 were available for testing for P2. The participant provided a written consent prior to study participation (IRB-16-01215).
Reporting on race, ethnicity, or other socially relevant groupings	The persistent infection index case and three related transmission cases were identified based on viral genomic surveillance of SARS-CoV-2 positive residual clinical specimens from the Mount Sinai Health System that were left over after conclusion of the diagnostic process, as part of approved protocols by the Mount Sinai Hospital Institutional Review Board.<br><br>Reference sera for neutralization assays were collected from participants in the longitudinal observational PARIS (Protection Associated with Rapid Immunity to SARS-CoV-2) study.
Population characteristics	The PARIS study follows a cohort of health care workers longitudinally since April 2020.
Recruitment	The samples were sourced from an ongoing observational study based on events of interest (booster vaccination or BA.1 breakthrough infection). We did not recruit participants specifically for the work presented in this manuscript
Ethics oversight	The study was reviewed and approved by the Mount Sinai Hospital Institutional Review Board (IRB-20-03374, IRB-16-01215, RB-22-00760, IRB-13-00981)

Note that full information on the approval of the study protocol must also be provided in the manuscript.

Field-specific reporting

Please select the one below that is the best fit for your research. If you are not sure, read the appropriate sections before making your selection.

☒ Life sciences ☐ Behavioural & social sciences ☐ Ecological, evolutionary & environmental sciences

For a reference copy of the document with all sections, see nature.com/documents/nr-reporting-summary-flat.pdf

Life sciences study design

All studies must disclose on these points even when the disclosure is negative.

Sample size	We used a total of 44 sera from 21 study participants to determine the neutralization activity of the different viruses. We had, at least, two longitudinal biospecimen available for each participant. The number of samples was determined based on an amount that allowed to perform robust statistical analyses, the number of donors and the ability to process samples. Biospecimen from both male and female study participants were included.
Data exclusions	No data were excluded.
Replication	Microneutralization assays were done once with each serum sample being serially diluted.
Randomization	Samples were assigned to different groups based on the previous history of SARS-CoV-2 infection and vaccination.
Blinding	All human samples were coded. Blinding was not an option since the longitudinal biospecimen from a given participant had to be run on the same microneutralization plate.

Reporting for specific materials, systems and methods

We require information from authors about some types of materials, experimental systems and methods used in many studies. Here, indicate whether each material, system or method listed is relevant to your study. If you are not sure if a list item applies to your research, read the appropriate section before selecting a response.

Materials & experimental systems

n/a	Involved in the study
☐	☒ Antibodies
☐	☒ Eukaryotic cell lines
☒	☐ Palaeontology and archaeology
☒	☐ Animals and other organisms
☒	☐ Clinical data
☒	☐ Dual use research of concern
☒	☐ Plants

Methods

n/a	Involved in the study
☒	☐ ChIP-seq
☒	☐ Flow cytometry
☒	☐ MRI-based neuroimaging

Antibodies

Antibodies used	mAb 1C7C7 Center for Therapeutic Antibody Development at The Icahn School of Medicine at Mount Sinai ISMMS (Millipore Sigma, Cat# ZMS1075)<br>HRP-conjugated streptavidin (Thermo Fisher Scientific, Cat# N100)
Validation	All commercial antibodies were validated by their manufacturers and were titrated in the lab to determine optimal concentration for experimentation. In-house biotinylated 1C7C7 monoclonal antibody was validated in cells infected with WT SARS-CoV-2, BA.1 and BA.1.23 viral isolates. MAb concentrations were standardized based on the assay and starting concentration is described in methods section.

Eukaryotic cell lines

Policy information about cell lines and Sex and Gender in Research

Cell line source(s)	Vero-E6-TMPRSS2 (BPS Biosciences, catalogue (cat.) no. 78081) and Vero-E6 cells-TMPRSS2-ACE2(BEI Resources, catalog (cat.) no. NR-54970)
Authentication	Cell lines were authenticated by supplier. No other authentication at the lab level was performed.
Mycoplasma contamination	Mycoplasma free, tested by the PCR-based assay Universal Mycoplasma Detection Kit (ATCC, Cat. 30-1012K).
Commonly misidentified lines (See ICLAC register)	Not commonly misidentified cell lines were used in this study.
